# Excessive Neutrophils and Neutrophil Extracellular Traps in COVID-19

**DOI:** 10.3389/fimmu.2020.02063

**Published:** 2020-08-18

**Authors:** Jun Wang, Qian Li, Yongmei Yin, Yingying Zhang, Yingying Cao, Xiaoming Lin, Lihua Huang, Daniel Hoffmann, Mengji Lu, Yuanwang Qiu

**Affiliations:** ^1^Center of Clinical Laboratory, The Fifth People's Hospital of Wuxi, Jiangnan University, Wuxi, China; ^2^Bioinformatics and Computational Biophysics, University of Duisburg-Essen, Essen, Germany; ^3^Institute of Virology, University Hospital of Essen, University of Duisburg-Essen, Essen, Germany; ^4^Department of Laboratory Medicine, Maternal and Child Health Hospital of Hubei Province, Wuhan, China; ^5^Radiology Department, The Fifth People's Hospital of Wuxi, Jiangnan University, Wuxi, China; ^6^Department of Infectious Diseases, The Fifth People's Hospital of Wuxi, Jiangnan University, Wuxi, China

**Keywords:** coronavirus, COVID-19, neutrophil extracellular trap, pneumonia, neutrophilia, lymphopenia

## Abstract

**Background:** Cases of excessive neutrophil counts in the blood in severe coronavirus disease (COVID-19) patients have drawn significant attention. Neutrophil infiltration was also noted on the pathological findings from autopsies. It is urgent to clarify the pathogenesis of neutrophils leading to severe pneumonia in COVID-19.

**Methods:** A retrospective analysis was performed on 55 COVID-19 patients classified as mild (*n* = 22), moderate (*n* = 25), and severe (*n* = 8) according to the Guidelines released by the National Health Commission of China. Trends relating leukocyte counts and lungs examined by chest CT scan were quantified by Bayesian inference. Transcriptional signatures of host immune cells of four COVID19 patients were analyzed by RNA sequencing of lung specimens and BALF.

**Results:** Neutrophilia occurred in 6 of 8 severe patients at 7–19 days after symptom onset, coinciding with lesion progression. Increasing neutrophil counts paralleled lesion CT values (slope: 0.8 and 0.3–1.2), reflecting neutrophilia-induced lung injury in severe patients. Transcriptome analysis revealed that neutrophil activation was correlated with 17 neutrophil extracellular trap (NET)-associated genes in COVID-19 patients, which was related to innate immunity and interacted with T/NK/B cells, as supported by a protein–protein interaction network analysis.

**Conclusion:** Excessive neutrophils and associated NETs could explain the pathogenesis of lung injury in COVID-19 pneumonia.

## Introduction

As of early May 2020, more than 3 million cases of coronavirus disease 2019 (COVID-19) have been confirmed worldwide, resulting in hundreds of thousands of deaths (1). According to the Guidelines of the Diagnosis and Treatment of New Coronavirus Pneumonia (version 7) published by the National Health Commission of China, COVID-19 patients can be classified as mild, moderate, and severe cases. Severe patients easily develop acute respiratory distress syndrome (ARDS) or multiple organ failure, with a 4–15% death rate (2, 3)

It is not well-understood what drives the exacerbated host response involving a cytokine storm in severe COVID-19 (4). Specifically, it is unclear what initiates and propagates the cytokine storm. Neutrophil infiltration was noted in three recent reports on the pathological findings from autopsied COVID-19 patients (5–7). Neutrophil infiltration in pulmonary capillaries, acute capillaritis with fibrin deposition, extravasation of neutrophils into the alveolar space, and neutrophilic mucositis were observed. Similarly, increased neutrophil counts were reported to occur simultaneously in the peripheral blood of severe and non-surviving COVID-19 patients (3, 8). Neutrophilia predicts poor outcomes in patients with COVID-19, and our previous research also indicated the neutrophil-to-lymphocyte ratio (NLR) is an independent risk factor for severe disease (8, 9).

Recently, two serum markers of neutrophil extracellular traps (NETs), myeloperoxidase (MPO)-DNA, and citrullinated histone H3 (Cit-H3) levels were found to be elevated in the serum of COVID-19 patients (10). This suggested that neutrophilia and excessive NETs may contribute to cytokine release and respiratory failure. As a contributor to pathological inflammation of pneumonia, excessive neutrophils lead to tissue injury by oxidative burst, phagocytosis, and the formation of neutrophil NETs, known as NETosis. NETs are composed of extracellular webs of DNA, histones, microbicidal proteins, and oxidative enzymes that are released by neutrophils to corral infections (11–15). The ability of NETs to damage tissues is well-documented in infection and sterile disease. NETs directly kill epithelial and endothelial cells (16, 17), and excessive NETosis damages the epithelium in pulmonary fungal infection (18) and the endothelium in transfusion-related acute lung injury (19).

In the present study, first, the dynamics of neutrophil counts in COVID-19 patients (*n* = 23) during hospitalization were examined, together with the corresponding lung injury, to clinically define the relationship between lung injury and leukocyte counts. Second, transcriptional signatures of host immune cells from COVID-19 patients (*n* = 4) were analyzed by RNA sequencing of lung specimens or bronchoalveolar lavage fluids (BALF). Immune cell frequency was analyzed by MCPcouter. We used average expression of genes enriched in neutrophil degranulation and activation to screen highly correlated genes and further identified NET associated genes in the correlated gene list to construct an interactive network from the STRING database.

## Methods

### Participants and Study Design

The study was approved by the Ethics Committee of the Fifth People's Hospital, Wuxi (No. 2020-006-1). The 55 confirmed COVID-19 patients were enrolled in this retrospective study from January 23 to March 15, 2020. Written informed consent was obtained from all patients from the Fifth People's Hospital, Wuxi, China.

The clinical handling of COVID-19 patients was performed according to the Guidelines of the Diagnosis and Treatment of New Coronavirus Pneumonia (version 7) published by the National Health Commission of China. Mild, moderate, and severe cases were defined by the following conditions: (1) epidemiological history, (2) fever or other respiratory symptoms, (3) frequency of typical CT image abnormalities of viral pneumonia, and (4) positive RT-PCR result for SARS-CoV-2 RNA. In addition, mild cases were diagnosed if no typical CT image abnormality of viral pneumonia (#3 above) was seen and severe patients also met at least one of the following conditions: (1) shortness of breath, respiratory rate (RR) ≥30 times/min, (2) oxygen saturation (resting state) ≤ 93%, or (3) PaO_2_/FiO_2_ ≤ 300 mm Hg.

### Data Collection

All medical records including epidemiological, demographic, clinical manifestation, laboratory data, radiological characteristics, treatment, and outcome data were reviewed and collected. Laboratory confirmation of SARS-CoV-2 infection was performed by real-time RT-PCR (Bojie Ltd, 119 Shanghai, China) according to Chinese CDC approval. Five sets of RNA-seq data from BALF of two COVID-19 patients were acquired from BIG Data Center (accession number CRA002390), and corresponding data of three healthy controls were from the NCBI SRA database (accession numbers SRR10571724, SRR10571730, and SRR10571732). Four RNA-seq data from lung specimens of two COVID-19 patients and two healthy controls were acquired from the GEO database (accession numbers GSM4462416, GSM4462415, GSM4462414, and GSM4462413).

### Chest CT Protocols

All images were obtained on the CT system (Somatom Definition AS+, Siemens Healthineers, Germany) with patients in supine position. The main scanning parameters were as follows: tube voltage = 120 kV, automatic tube current modulation (about 95 mAs), pitch = 1.2 mm, slice thickness = 7 mm, field of view = 350 mm × 350 mm. All images were then reconstructed with a slice thickness of 0.6 mm with the same increment.

### Image Analysis

Two professional radiologists (Y.M.Y. and X.M.L.), who were blinded to the laboratory test data, reported chest CT features and assessed the CT features by consensus. The lesion CT values were assessed using the Skyview pacs system. The region-of-interest was selected manually marking the area of highest intensity (most restricted area) of the lesion in CT images.

### RNA-Seq Library Sequencing and Analysis

Kallisto was used to pseudoalign the RNA-seq reads and perform bootstrap analysis using an index based on the ENSEMBL GRCh38 *Homo sapiens* release 99 transcriptomes (20). Gene expression levels were then calculated as transcripts per million (TPM). Sleuth (version 0.30.0) (21) was used to perform differential gene expression (DEGs) analysis with the Wald test. Benjamini-Hochberg-adjusted false discovery rate (*q* < 0.1) was used to correct for multiple comparisons.

To compare lung and BALF samples of COVID-19 patients with healthy controls, differentially expressed genes were exhibited in a scaled heatmap using pheatmap (22). MCP-counter was used to characterize immune cell subpopulations (23). The MCP-counter scores obtained from the three underlying transcriptome platforms (Affymetrix Human Genome U133 Plus 2.0, Affymetrix 133A, and Illumina HiSeq) were used to estimate the expression of each cell population. Functional enrichment analysis of the 29 upregulated marker genes of neutrophils was conducted with Metascape (http://metascape.org/) (24). Gene set enrichment analysis (GSEA) was performed in pre-ranked list mode with 1,000 permutations and weighted enrichment statistic (25). The gene interaction was analyzed by STRING (26). Gene interaction networks were visualized with eXamine (27).

### Statistical Analyzes

Quantitative parameters are described as the median value followed by the inter-quartile range (IQR) in parentheses. Principal component analysis was performed with R package “FactoMineR” to identify those clinical parameters that contribute most to distinguishing severe, moderate, and mild cases of COVID-19 (28). Figures were produced with R package “ggplot2” (29). Logistic regression was conducted with R package “rstanarm” (30) to identify associations of laboratory parameters with severity of cases.

Severe cases were typed as severe and others (moderate and mild cases) as non-severe. The generalized linear model was then used to calculate coefficients (mean value with 5%, 95% confidence interval) of all parameters for severe. Finally, we used the function of exp [exp(x) = ex] for coefficients. The results were an odd's ratio (mean, 5–95% credible interval). Receiver operating characteristic curves (ROC) were calculated by R package “pROC.” The area under the ROC curve (AUC) and cut-off values of selected parameters were used to distinguish mild and severe cases (31). Numerical Bayesian linear regression was carried out with Stan using Hamiltonian Monte Carlo (Supplemental Materials; Supplementary Figure 1) (32).

## Results

### Characteristics of COVID-19 Patients

Fifty-five confirmed COVID-19 patients were hospitalized in The Fifth People's Hospital of Wuxi from Jan 23 to Mar 15, 2020. The median age of patients was 45 years (IQR 25–61), and 27 (49%) were male. Based on the previously described guidelines, 22 (40%), 25 (45%), and 8 (15%) of the 55 COVID-19 patients were classified as mild, moderate, and severe cases, respectively. There were five patients with diabetes (9%), 13 with hypertension (24%), eight with surgical history (15%), and two with co-infections (4%). The most common symptoms at onset were fever in 28 cases (51%), sputum production in 13 cases (24%), cough in 22 cases (40%), and fatigue in 17 cases (31%) (Table 1).

**Table 1:** Demographic and clinical characteristics of 55 COVID-19 patients.

**Variable**	**Value**
Age (year)	45.0 (25.0–61.0)
Gender—no./(%)	
Male	27 (49.1)
Female	28 (50.9)
Clinical diagnosis—no./(%)	
Severe	8
Moderate	25
Mild	22
Initial symptoms—no./(%)	
Fever (>38°C)	28 (50.9)
Sputum production	13 (23.6)
Headache	5 (9.1)
Chill	7 (12.7)
Shivering	2 (3.6)
Nausea or vomiting	1 (1.8)
Diarrhea	13 (23.6)
Fatigue	17 (30.9)
Cough	22 (40)
Pharyngalgia	2 (3.6)
Rhinorrhea	6 (10.9)
Chest pain	1 (1.8)
Shortness of breath	5 (9.1)
Chest tightness	9 (16.4)
Chronic disease—no./(%)	
Diabetes	5 (9.1)
Hypertension	13 (23.6)
Thyroid disease	2 (3.6)
Malignant tumor	2 (3.6)
Gastritis	2 (3.6)
Coronary artery disease	1 (1.8)
Surgical history	8 (14.5)
Co-infection—no./(%)	
Initial	0
Progressive	2 (3.6)

**Figure 1:**
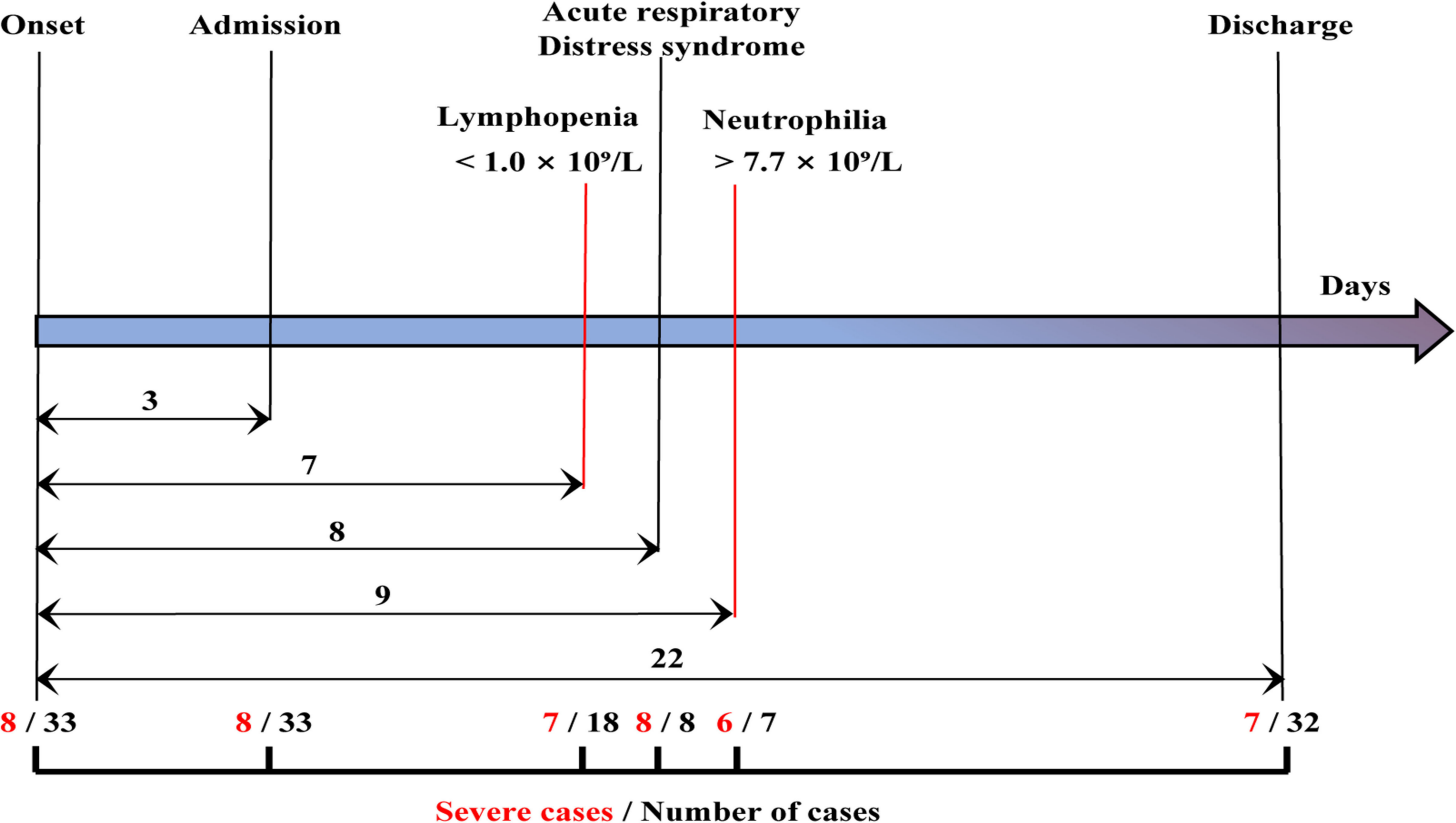
Clinical courses of the study patients. The time lines showed the days of hospital admission, lymphopenia, acute respiratory distress syndrome, neutrophilia, and discharge from symptom onset for each case. The median time from onset of symptoms to hospital admission and discharge was 3, 22 days, respectively. Among the 33 patients (black), eight cases were severe cases (red). A total of 18 cases exhibited lymphopenia within 7 days, including seven severe patients. All eight patients presented with acute respiratory distress syndrome within 8 days. Seven cases presented with neutrophilia, including six severe cases within 9 days.

The clinical handling and relevant time-points of 33 patients including eight severe and 25 moderate cases are shown in Figure 1. The median time from the date of onset of symptoms to hospital admission, lymphopenia, ARDS, and neutrophilia was 3, 7, 8, and 9 d, respectively. Lymphopenia occurred in seven of eight severe patients and 11 of 25 moderate cases within 7 d, ARDS occurred in all eight severe patients within 8 d, and neutrophilia occurred in six of eight severe patients and one of 25 moderate cases within 9 d (Figure 1).

The laboratory test of each patient on the day of hospital admission showed that the median neutrophil count in severe COVID-19 patients (3.4, IQR: 1.8–6.7) was higher than in the moderate (3.0, 2.4–3.6) and mild (2.9, 2.3–3.5) groups. In contrast, lymphocyte and monocyte counts in severe COVID-19 patients were lower than in the other two groups (Table 2). By logistic regression, the following ORs for effects on having a severe case were obtained: neutrophil counts (1.5, 95% CI: 1.0–2.1), ratio of neutrophil to lymphocyte (NLR; 1.2, 95% CI: 1.1–1.4), C-reactive protein (CRP; log-scaled; 2.6, 95% CI: 1.6–4.7), Fibrinogen (FIB, 2.6, 95% CI: 1.5–4.9), and thrombin time (TT, 2.5, 95% CI: 1.4–5.0). These findings suggest that higher neutrophil counts, the NLR, and CRP, FIB, and TT levels as potential prognostic factors. The ORs of lymphocyte (0.28, 95% CI: 0.08–0.85) and monocyte (0.02, 95% CI: 0.00–1.16) counts suggest an association of lower lymphocyte and monocyte counts with severe pneumonia.

**Table 2:** Laboratory parameters of mild, moderate, and severe COVID-19 cases.

**Baseline variables**	**Reference range**	**Severe cases (*n* = 8)**	**Moderate cases (*n* = 25)**	**Mild cases (*n* = 22)**	^*^Odds ratio for severe (95% CI)
Age (year)		59 (50–73)	45 (30–60)	39.5 (22.3–52)	1.07 (1.02–1.12)
Female (%)		3 (37.5)	11 (44)	14 (64)	1.90 (0.57–7.34)
**Blood routine**					
White blood cell (×10^9^/L)	3.5–9.5	5.4 (3.4–7.6)	4.8 (4.1–5.7)	5.3 (4.7–6.8)	1.23 (0.86–1.78)
Neutrophil (×10^9^/L)	1.8–6.3	3.4 (1.8–6.7)	3.0 (2.4–3.6)	2.9 (2.3–3.4)	1.47 (1.05–2.14)
Lymphocyte (×10^9^/L)	1.1–3.2	1.0 (0.7–1.6)	1.3 (0.9–1.5)	1.9 (1.1–2.8)	0.28 (0.08–0.85)
Monocyte (×10^9^/L)	0.1–0.6	0.4 (0.2–0.6)	0.5 (0.4–0.6)	0.5 (0.4–0.6)	0.02 (0.00–1.16)
Platelet (×10^9^/L)	125.0–350.0	154.0 (121.0–182.8)	191.0 (156.5–213.5)	194.5 (163.8–214.5)	0.98 (0.97–1.01)
PDW (CV %)	15.5–18.1	15.4 (11.4–16.6)	14.2 (13.7–15.9)	12.8 (11.1–14.0)	1.12 (0.83–1.50)
Red blood cell (×10^12^/L)	4.30–5.80	4.3 (4.0–4.9)	4.8 (4.1–5.0)	4.4 (4.0–4.7)	0.48 (0.15–1.50)
RDW (CV %)	11.5–14.9	12.9 (12.1–13.9)	12.4 (11.7–13.6)	11.9 (11.6–12.3)	1.91 (1.19–3.41)
Ratio of neutrophils to lymphocytes		2.4 (1.4–16.2)	2.3 (1.7–2.9)	1.8 (0.9–2.8)	1.21 (1.06–1.42)
Ratio of monocytes to lymphocytes		0.3 (0.3–0.8)	0.4 (0.2–0.5)	0.3 (0.2–0.4)	2.86 (0.28–27.0)
C-reactive protein (mg/L)	0.0–10.0	41.1 (13.8–139.9)	6.2 (1.1–12.7)	2.1 (0.5–17.7)	2.64 (1.64–4.65)
**Biochemical indicators**					
ALT (U/L)	4.0–44.0	17.0 (14.0–60.0)	19.0 (16.0–35.3)	26.0 (14.0–43.3)	1.02 (0.99–1.04)
AST (U/L)	8.0–38.0	28.0 (23.0–49.0)	23.5 (20.8–31.3)	24.5 (19.0–31.0)	1.04 (0.99–1.09)
Total bilirubin (μmol/L)	2.0–21.0	7.0 (3.0–12.0)	5.0 (2.8–9.0)	6.5 (4.8–10.3)	1.01 (0.87–1.16)
Direct bilirubin (μmol/L)	2.0–7.0	0.1 (0.1–1.0)	0.1 (0.1–1.8)	0.1 (0–1.4)	0.99 (0.67–1.37)
Serum total protein (g/L)	67.0–83.0	68.0 (65.0–75.0)	69.5 (65.0–73.3)	69.0 (65.0–71.3)	0.98 (0.87–1.16)
Serum albumin (g/L)	35.0–50.0	39.0 (34.0–43.0)	43.5 (38.8–47.3)	41.5 (38.8–45.0)	0.85 (0.73–1.00)
Creatine kinase (U/L)	0.0–171.0	101.0 (54.0–151.0)	69.0 (53.8–106.8)	68.0 (46.8–102.0)	1.000 (0.99–1.01)
Creatine kinase MB (U/L)	0.0–12.0	11.0 (10.0–13.0)	10.0 (9.0–12.5)	10.0 (7.8–14.8)	0.97 (0.79–1.17)
Blood urea nitrogen (mmol/L)	3.1–8.0	5.9 (3.3–10.1)	4.2 (3.5–4.9)	4.0 (3.0–4.6)	1.60 (1.18–2.31)
Serum creatinine (μmol/L)	53.0–97.0	64.0 (38.0–88.0)	54.5 (43.5–64.5)	48.5 (39.3–58.5)	1.04 (1.01–1.08)
Serum potassium (mmol/L)	3.8–5.0	3.8 (3.2–4.2)	4.1 (3.8–4.2)	4.0 (3.9–5.0)	0.24 (0.06–0.79)
Serum sodium (mmol/L)	136.0–149.0	140.0 (13.9.0–141.0)	142.0 (141.0–143.0)	142.0 (140.0–143.0)	0.59 (0.38–0.89)
Serum chlorine (mmol/L)	98.0–106.0	105.0 (103.0–106.0)	104.0 (102.8–106.0)	105.0 (103.0–106.0)	0.72 (0.95–1.25)
**Blood coagulation function**					
D-dimer (mg/L)	0.0–0.5	0.6 (0.3–1.2)	0.3 (0.2–0.6)	0.3 (0.2–0.5)	1.314 (0.579–2.986)
PT (s)	11.5–15.5	13.2 (12.2–13.4)	13.2 (12.9–13.6)	13.2 (13.2–13.5)	0.22 (0.05–0.90)
APTT (s)	26.0–40.0	37.5 (35.5–42.3)	38.2 (36.3–42.9)	41.3 (37.6–44.3)	0.93 (0.79–1.09)
Fibrinogen (g/L)	2.0–4.0	4.9 (4.4–5.9)	3.6 (2.9–4.8)	3.6 (2.7–4.1)	2.61 (1.52–4.87)
TT (s)	14.0–21.0	17.1 (16.2–18.2)	16.2 (15.8–16.8)	16.2 (15.9–17.4)	2.46 (1.35–4.97)
**Blood gas analysis**					
PaCO_2_ (mm Hg)	35.0–48.0	42.5 (39.3–44.0)	43.0 (40.5–47.0)	42.0 (40.3–45.0)	0.92 (0.76–1.09)
PaO_2_ (mm Hg)	83.0–108.0	83.0 (64.5–00.5)	106.0 (93.5–134.0)	103.5 (93.3–124.3)	0.95 (0.91–0.98)
PaO_2_/FiO_2_ (mm Hg)	400.0–500.0	395.2 (300.0–478.6)	504.8 (445.2–632.6)	461.9 (395.6–591.7)	0.99 (0.98–1.00)
Lactic acid (mmol/L)	0.5–2.2	1.9 (1.3–3.4)	1.6 (1.3–1.9)	1.7 (1.1–2.3)	2.44 (1.07–5.93)

* *The Odd Ratio of log normalization*.

### Principal Component Analysis and Dynamic Monitoring of Laboratory Parameters

**Figure 2:**
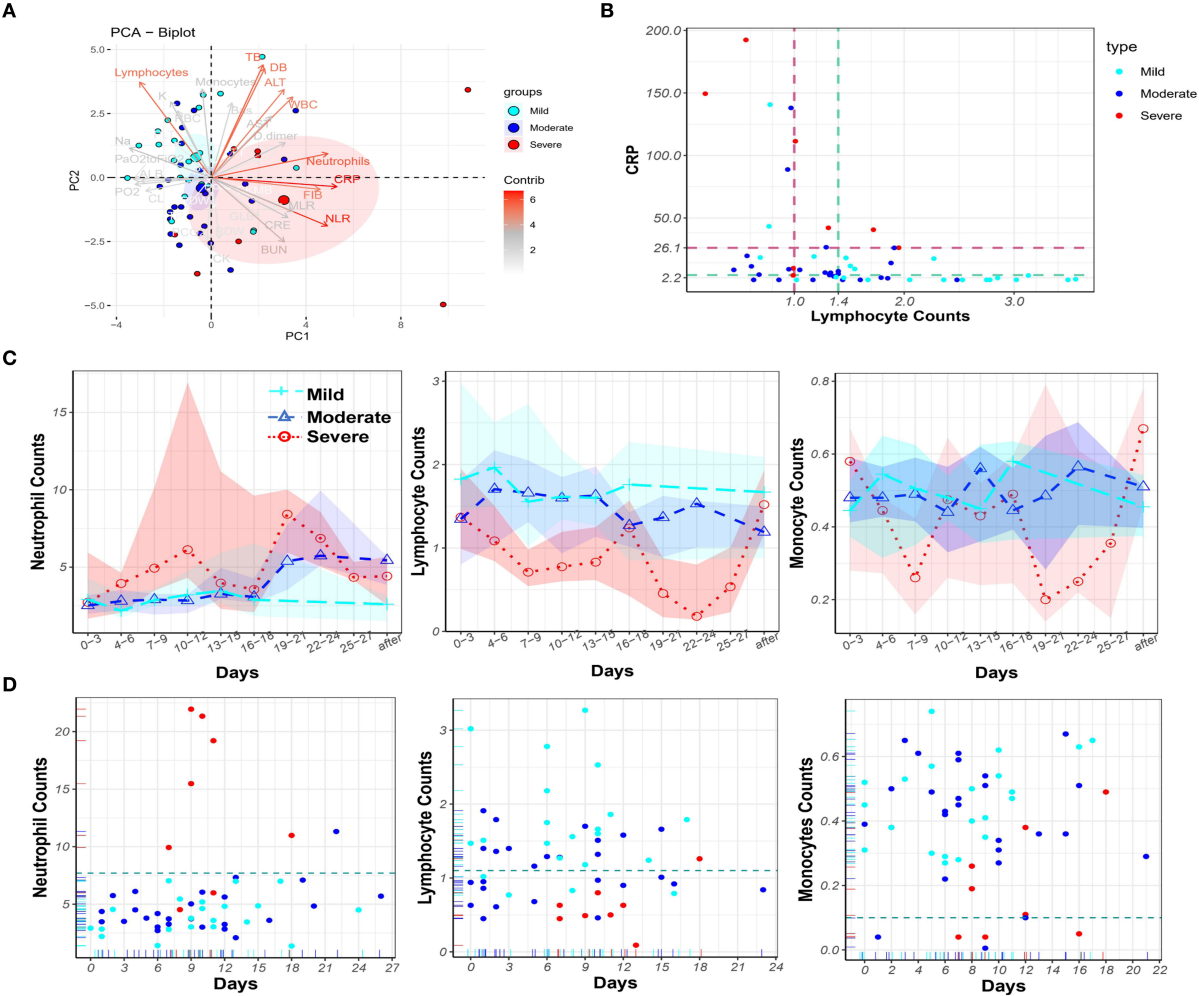
Principal component analysis of laboratory parameters and dynamic monitoring of blood cells in the peripheral blood of COVID-19 patients. **(A)** Principal component analysis to identify variables for distinguishing the disease severity of COVID-19 patients. The nine variables that contributed mostly to distinguishing the disease severity were white blood cell counts (WBC), neutrophil counts, neutrophil-to-lymphocyte ratio (NLR), C-reactive protein (CRP), FIB, ALT, Total Bilirubin (TB), Direct Bilirubin (DB), and lymphocyte counts. **(B)** CRP levels and lymphocyte counts in 55 cases with the cut-off values CRP = 26.1 and lymphocyte = 1.0 for eight severe cases (brown) and the cut-off values CRP = 4.3 and lymphocyte = 1.4 for 22 mild cases (green). **(C)** The dynamic change of neutrophil, lymphocyte and monocyte counts over time in COVID-19 patients in mild (cyan), moderate (blue), and severe (red) groups, circled dots: mean value; colored background area: IQR (interval quartile range). The **(D)** Time points of maximum neutrophil, minimum lymphocyte, and minimum monocyte counts, and the corresponding counts in mild (cyan), moderate (blue), and severe (red) COVID-19 patients during hospitalization.

Principal component analysis was performed to visualize the contribution of all mentioned clinical parameters on disease severity (Figure 2A). Nine variables contributed most strongly. Among them, higher CRP, FIB, neutrophil count, and NLR, and lower lymphocyte count were associated with increased disease severity. These parameters may therefore be used for prognosis. To assess the diagnostic value of the top two contributors, CRP and lymphocytes, the AUC and cut-off values from the ROC curves were calculated for the severe and mild cases, respectively (Supplementary Figure 2B). The cut-off values for severe patients were CRP (26.1) and lymphocytes (1.0), and for mild patients the values were CRP (2.2) and lymphocytes (1.4) (see dashed lines in Figure 2B).

Next, dynamic changes of neutrophil, lymphocyte, and monocyte counts in the peripheral blood of COVID-19 patients were monitored (Figure 2C). Dramatically increased neutrophil counts were found in severe COVID-19 patients in comparison to the other two groups. In contrast, lymphocyte counts persisted at lower values in severe COVID-19 patients. Monocyte counts were lower in severe cases, although the monocyte count fluctuated over a wide range. Timing of the occurrence of maximum neutrophil, minimum lymphocyte, and minimum monocyte counts, and the corresponding counts in COVID-19 patients, during hospitalization are shown in Figure 2D. From day 7 to day 9 after symptom onset, neutrophil counts erupted (>7.7 × 10^9^/L) and peaked in six of eight severe COVID-19 patients. In contrast, only one moderate (1/26) COVID-19 patient was found with neutrophilia. Lymphopenia occurred in seven of eight severe patients but only in four mild (4/22) COVID-19 patients. Monopenia (<1 × 10^8^/L) was found in three moderate (3/25) and four severe (4/8) COVID-19 patients. Overall, monitoring blood cell parameters revealed neutrophilia as a characteristic of severe COVID-19 patients.

### Bayesian Linear Regression of CT Values and Changing Neutrophil and Lymphocyte Counts

**Figure 3:**
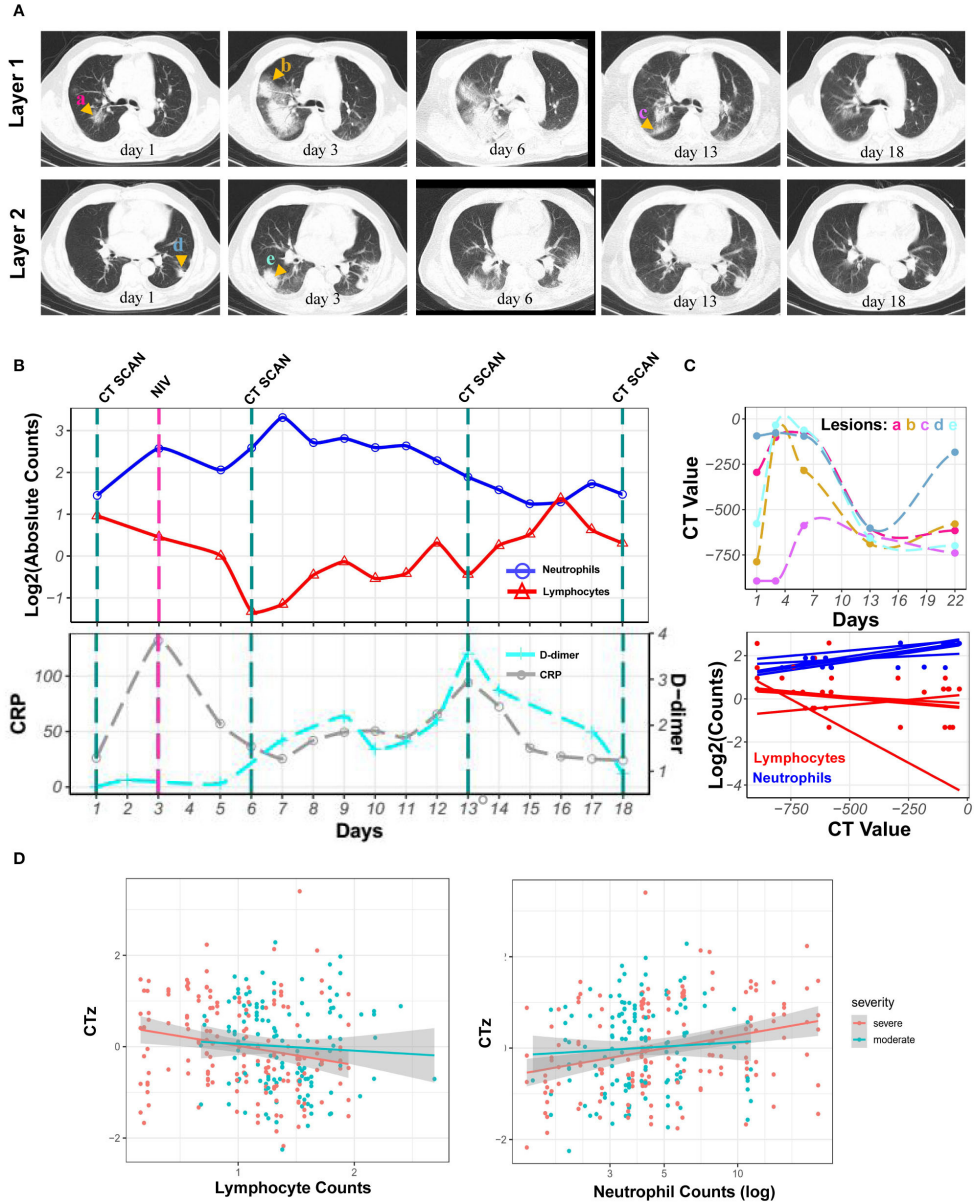
Kinetics of laboratory parameters and serial chest CT images of severe COVID-19 patient with the development of neutrophilia. **(A)** Normal chest CT with axial planes at indicated time point. **(B)** The dynamics of neutrophil counts (blue line), lymphocyte counts (red line) with log_2_ scaling, and CRP (gray line) and D-dimer (cyan line) levels at indicated time point. **(C)** CT value of lesions and its correlation with log_2_ scaled neutrophil and lymphocyte counts at indicated time point. **(D)** Least-square fits of linear models to summarize the z-values of CT values as a function of log-transformed neutrophil counts for 23 patients. Points are pairs of CTz values (*z*-values of individual CT measurements) and log-neutrophil counts, colored according to severity of COVID-19. Colored lines are the corresponding least-square fits to the data form each severity group. Gray areas are 95% confidence intervals.

Neutrophilia and lymphopenia obviously occurred in severe COVID-19 patients during hospitalization. Here was a case of severe patient. The CRP level remained low when neutrophilia occurred, and the D-dimer levels increased after neutrophilia. Series of chest CT images exhibited enlarged patches and ground-glass nodules in the sub-pleura area of both lungs during neutrophilia. Interestingly, all observed lesions were reduced or gradually absorbed along with the return of neutrophils to normal levels after neutrophilia (Figures 3A,B). The CT value of lesions, reflecting lung lesions, was further demonstrated to have the same trend with neutrophils but the opposite trend with lymphocytes (Figure 3C).

To estimate the overall correlation of CT value with neutrophil and lymphocyte counts across patients with a visual inspection of possible trends, linear models were fitted to summarize the dependency of *z*-values of CT value (CTz, see Supplementary Information) of neutrophil and lymphocyte counts. Thus, Bayesian linear regression was used to quantify the observed trends of CTz values as a function of parameters mentioned above. For log-transformed neutrophil counts, a slope for the moderate cases of 0.3 [−0.3, 0.9] (0.05 and 0.95 quantiles in square brackets) was obtained, i.e., with a slope that could be flat. For the severe cases, the mean slope was 0.8 [0.3, 1.2], i.e., clearly positive. Thus, no clear trend for moderate cases was visible, whereas an increase in CTz value with neutrophil counts was significantly correlated for severe cases. For CTz as a function of lymphocyte counts, the slope was −0.1 [−0.4, 0.6] for moderate cases and −0.3 [−0.5, 0.0] for the severe cases, supporting the trends in Figure 3D. Overall, the results showed that the CTz value has no average trend with changing neutrophil and lymphocyte counts for moderate cases (green). However, for the severe cases (red), there are clear trends for CTz value with changing cell counts; specifically, CTz value increased for increasing neutrophil counts, whereas CTz value decreased for increasing lymphocyte counts (Figure 3D).

### Immune Cell Transcriptional Signatures of the Lung and BALF in COVID-19 Patients

**Figure 4:**
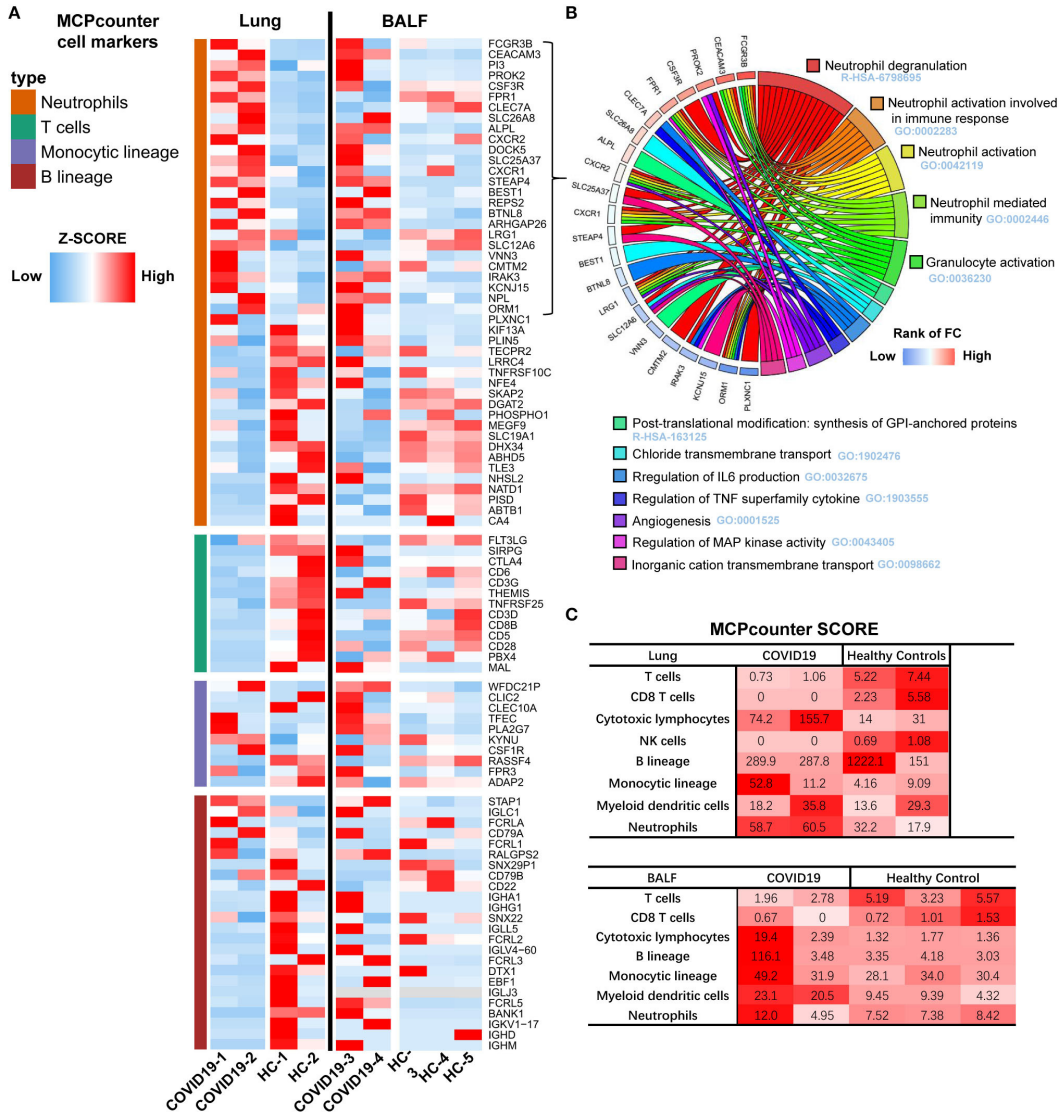
Transcriptome analysis of the lung and BALF in COVID-19 patient. **(A)** Marker genes from Microenvironment Cell Populations-counter (MCP-counter) were used to identify of Neutrophils, T cells, Monocytes, and B cells in both Lung and BALF samples of COVID-19 patients and healthy controls, respectively. The RNA-seq data TPM are shown in a scaled heatmap. **(B)** Circle plots for functional enrichment analysis of 29 marker genes of Neutrophils. **(C)** The absolute abundance of immune cell subpopulations as scores in COVID-19 patients and healthy cases.

Immune cell transcriptional signatures were established from RNA-seq data of BALF and lung specimens of COVID-19 patients and healthy controls. Marker genes of neutrophils, T cells, monocytes, and B cells were identified from Microenvironment Cell Populations-counter (MCP-counter). Their representation in the RNA-seq data were exhibited using a scaled heatmap by comparing both lung and BALF samples of COVID-19 patients to healthy controls (Figure 4A).

The results revealed that 112 marker genes represented four immune populations: neutrophils (46 genes), T cells (13 genes), monocytes (10 genes), and B cells (43 genes). For lung tissue, the most up-regulated marker genes were enriched in neutrophils, second in monocytes, and only a small proportion were enriched in B cells. Marker genes of T cells were almost all lowly expressed. For BALF, the most upregulated marker genes were similarly enriched in neutrophils, but more up-regulated genes in monocytes and B cells were observed in COVID-19 patients compared to healthy controls, which is different from the lung samples.

Functional enrichment analysis of the 27 upregulated marker genes of neutrophils were further conducted with Metascape. The enrichment analysis revealed that five gene sets with lowest *q*-value were related to neutrophil degranulation and activation (Figure 4B) and there were 15 marker genes involved. Then, we calculated the average expression of these genes as an evaluating score for neutrophil activation (NAS).

To further assess the abundance of infiltrating immune cells of the lung and BALF in COVID-19 patients, the MCP-counter score was used to quantify the absolute abundance of immune cell subpopulations. Notably, the neutrophil scores were higher and T cell scores were lower in lung samples of COVID-19 patients. The higher abundance of cytotoxic T lymphocytes contributed for cell injury, not for anti-virus. Due to the marker genes for cytotoxic T lymphocytes was *KLRC1* (Killer Cell Lectin Like Receptor C1). For the BALF samples, the score of neutrophils, cytotoxic lymphocytes, B cells, monocytes, and dendritic cells were found to be higher in one of the COVID-19 patients compared to the three healthy controls (Figure 4C).

### Neutrophil Activation Related Genes Enrichment Analysis

**Figure 5:**
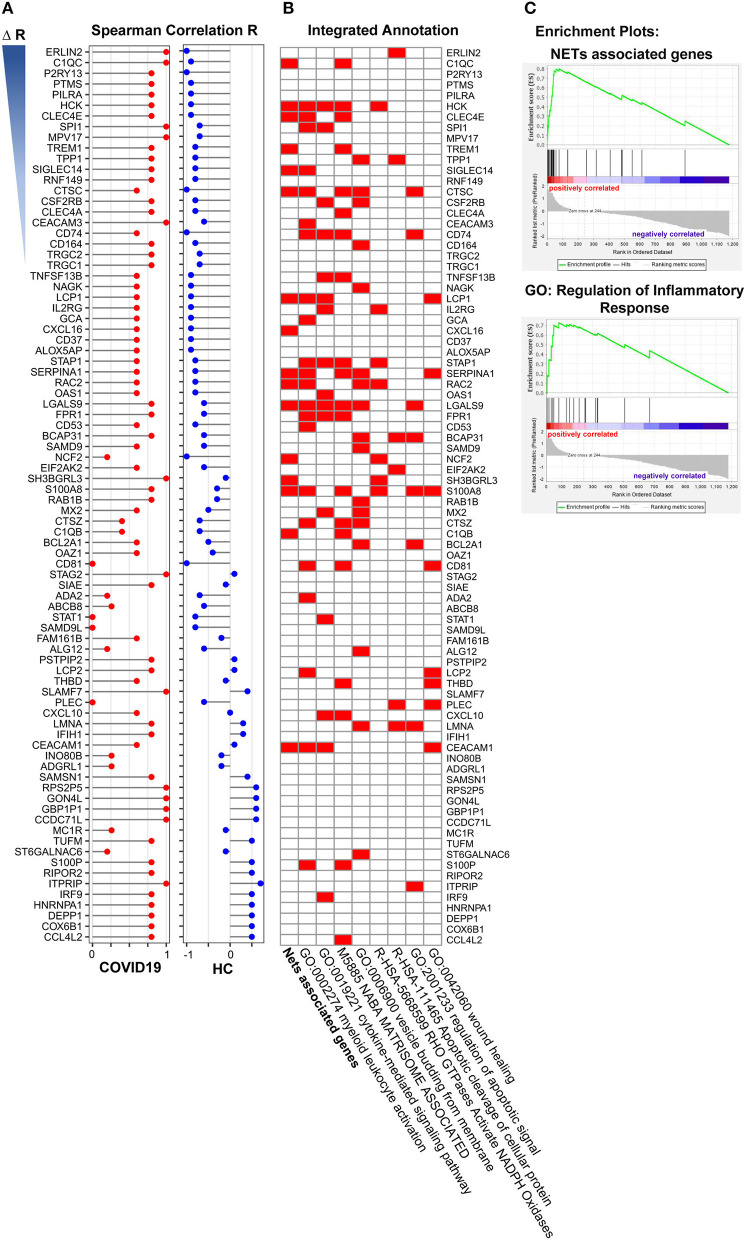
Gene enrichment analysis of neutrophil activation related genes. **(A)** The 15 annotated genes of neutrophils activation were calculated the average expression of every single samples as neutrophils activation score, and the correlation of the score with overlapped 1,363 differently expressed genes both in COVID-19 and Healthy control were analyzed. The selected 84 genes were ranked based on ΔR (R1-R2, R1 from COVID-19 patients, R2 from healthy control). **(B)** Functional enrichment analysis of these 84 genes, of which 16 genes were NETs associated genes. **(C)** Nets associated genes set (Enrichment Score, 0.80) and the GO term of regulation of inflammatory (Enrichment Score, 0.72) by GSEA with DEGs from pre-ranked by ΔR.

To explore the outcome of neutrophil activation in COVID-19, we further analyzed the correlation of NAS with 1,363 DEGs that overlapped in both the lung and BALF samples. The spearman correlation was used separately for COVID-19 patients and healthy controls. Then, the R value for every single gene was acquired for COVID-19 patients (R1) and healthy cases (R2). All DEGs were ranked based on ΔR (R1-R2). The “R value” of the top 84 genes (R1 > 0) in the two groups are displayed in Figure 5A. Of these 84 genes, 16 genes were NETs associated genes (Figure 5B; Table 3) (33–46) Of the 16 genes, *LGALS9, HCK, LCP1, CEACAM1* were involved in the cytokine-mediated signaling pathway. *S100A8, LGALS9*, and *CTSC* were involved in regulation of apoptotic signal by enrichment annotation from the Metascape tool (Figure 5B; Table 3) (33–46).

**Table 3:** Annotation of Nets associated genes.

**Function**	**Gene name**	**References**
Metabolic enzymes	RAC2; NCF2	(33, 34)
Structural proteins	LCP1	(35)
An-microbial related proteins	TREM1; S100A8; C1QB; C1QC	(35–37)
Peroxisomal enzyme	SH3BGRL3	(38)
Not classified	LGALS9; SERPINA1; CEACAM1; HCK; CXCL16; CLEC4E; CTSC; SIGLEC14	(39–46)

To further investigate the role of NETs in COVID-19, we generated a gene set termed “NET-associated genes” based on genes coding for proteins enriched in NETs released from human neutrophils with mass spectrometry (Supplementary Table 1). Pre-ranked GSEA by ΔR resulted in significant enriched gene sets of “NET-associated genes” (Enrichment Score = 0.80) and “Regulation of inflammatory response” (Enrichment Score = 0.72) (Figure 5C).

### NETs Associated Genes From RNA-Seq Data in COVID-19 Patients

**Figure 6:**
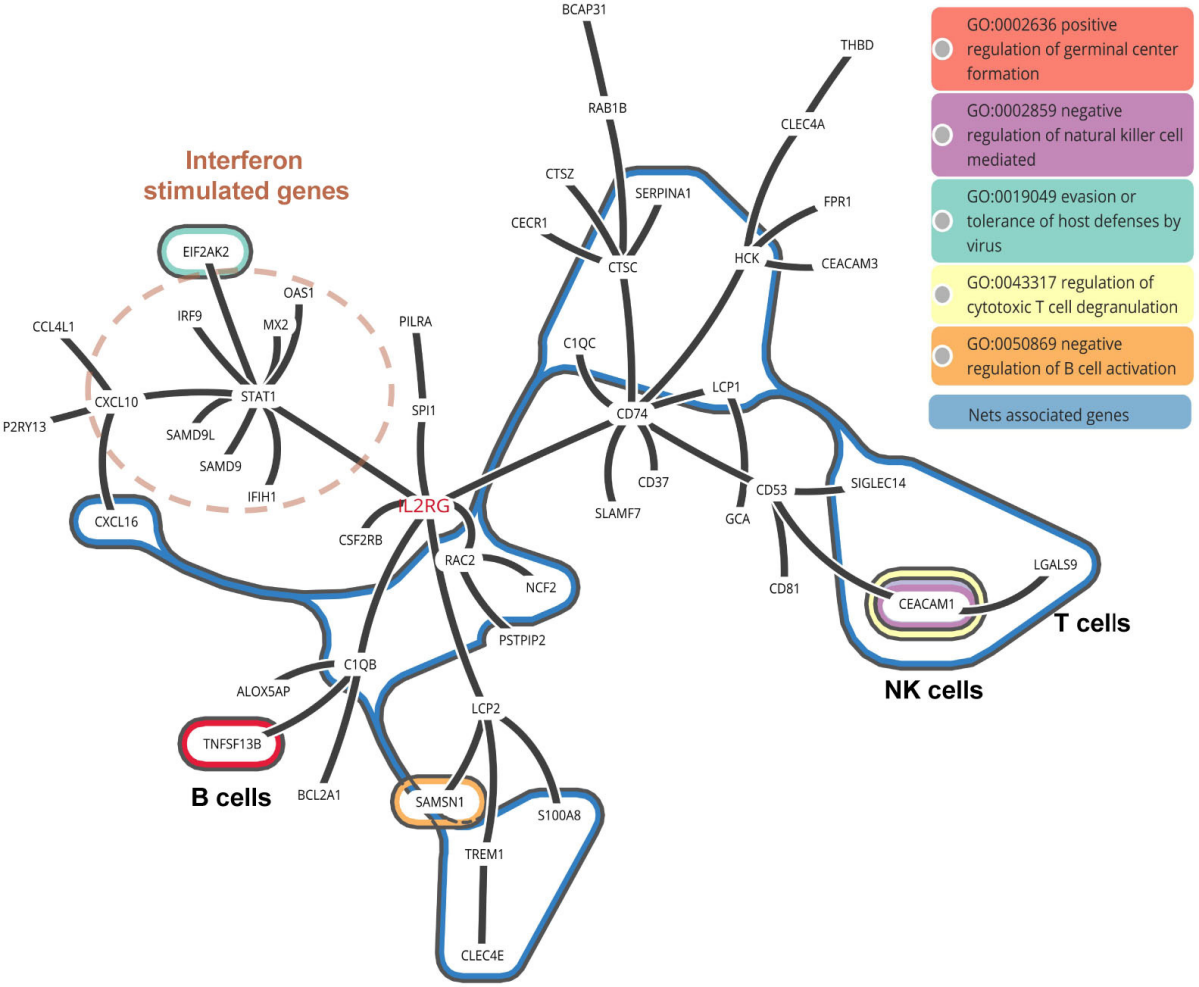
PPI network of NETs associated genes in COVID-19 patients. The interaction between NETs associated genes with other neutrophil activated genes.

As known, the formation of NETs could induce direct lung injury (17). There were 16 NETs associated genes related with neutrophils activation in COVID-19 patients. To further illustrate the interaction between these NETs associated genes with other neutrophils activation related genes, we constructed a protein-to-protein interaction network from the STRING database (Figure 6). We found that the NETs interacted with *STAT1* induced Interferon stimulated genes by *IL2RG*, implying that NETs associated genes may be triggered by IFN signaling. Besides, NETs in turn may activate B cells via *TNFSF13B* and inhibit the function of T and NK cells via *LGAS9* and *CEACAM1*, which are negative regulators for T and NK cells. *LGAS9* is a possible promoter of protein-arginine deiminase type 4 (PAD4). *PAD4*, a key NETs associated gene, lies downstream of ROS and promotes chromatin decondensation (47, 48). Of note, we also observed ROS related genes including *HCK, RAC2*, and *NCF2* among NETs associated genes (Figure 6).

To annotate the function of NETs associated genes, they were categorized as metabolic enzymes (*RAC2, NCF2*), structural proteins (*LCP1*), anti-microbial related (*TREM1*), peroxisomal (*SH3BGRL3*), and others (*C1QC, LGALS9, SERPINA1, C1QB, CCL7, CCL8, CEACAM1, HCK*, and *CXCL16*) (Table 3). Thus, we speculate that NETs may be activated by innate immunity such as IFN signaling, in COVID-19 patients. NETs may negatively regulate the immune function of T cells and NK cells via *LGAS9* and *CEACAM1*, respectively, leading to insufficient anti-viral immunity and injuring the lung tissue directly.

## Discussion

In this study, a set of laboratory test parameters and the corresponding chest CT images of 55 COVID-19 patients were collected during hospitalization. Among these variables, excessive neutrophils were associated with disease severity, as shown by principal component analysis. Bayesian inference across patients quantified that the increased trend of pneumonia lung injury, as represented by CT values, was in accord with the increased trend in neutrophil counts. Transcriptome analysis of lung specimens and BALF from COVID-19 patients also indicated the most up-regulated marker genes were neutrophil related. Importantly, many neutrophil activation genes were categorized as NET-associated genes. These genes were further assessed to interact with T and NK cells via negative regulatory molecules in COVID-19 patients leading to insufficient anti-viral response and lung injury (Figure 6).

Our previous study also found an increased neutrophil-to-lymphocyte ratio in the most severe disease cases (9). Recently, neutrophil infiltration was also noted in the lung tissue of autopsied COVID-19 patients (5–7). Since neutrophilia predicts poor outcomes in patients with COVID-19 (8), we propose that the change in neutrophil counts in peripheral blood or tissues may be closely associated with pathological injury in COVID-19 patients. We demonstrated here that the dynamics of neutrophil counts in COVID-19 patients during hospitalization exhibited the same trend as the corresponding lung injury.

NETs, as confirmed contributors to pathological inflammation of pneumonia, can damage tissues by killing epithelial and endothelial cells (16, 17) of pulmonary tissue in infection and sterile disease. Recently, two elevated NETs markers have been observed in serum from COVID-19 patients, which suggests that neutrophilia and excessive NETs may contribute to cytokine release and respiratory failure in COVID19 patients (10). However, evidence is still lacking regarding NETosis in lungs. We analyzed the differentially expressed genes in lung tissue and BALF samples from COVID-19 patient in comparison to healthy controls. Among all up-regulated genes in neutrophil modules in COVID-19 patients, we found 17 genes derived from the neutrophil activation pathway were NETs associated genes. Thus, NETs may be activated in the lung of COVID-19 patients. It is also poorly understood how NETosis induces the cytokine storm or modulates the host immune response. Our STRING analysis suggests that NETs associated genes could interact with T, NK, and B cells through regulation of *LGALS9, CEACAM1*, and *TNFSF13B* expressions, respectively. We suspect that the progression of lesions in COVID-19 patients may be induced by NETs as well as NETs-T/NK/B cell interactions.

In conclusion, the clear trend of lung injury in accord with the trend of increasing neutrophils was quantified by Bayesian inference analysis in COVID-19 patients. The transcriptome signature of immune cells also indicated elevated neutrophil markers in the lung and BALF samples of COVID-19 patients. Importantly, among the excessive neutrophil activated genes, 17 were NETs associated genes and these genes interacted with T cells and NK cells through negative regulation. Therefore, we posit that NETosis in lung tissue leads to an insufficient anti-viral response in COVID-19 patients. We hope that future studies will investigate the predictive power of circulating NETs in well-phenotyped longitudinal cohorts.

## Data Availability Statement

The datasets presented in this study can be found in online repositories. The names of the repository/repositories and accession number(s) can be found in the article/Supplementary Material.

## Ethics Statement

The studies involving human participants were reviewed and approved by the Ethics Committee of the Fifth People's Hospital, Wuxi (No. 2020-006-1). The patients provided their written informed consent to participate in this study.

## Author Contributions

JW, YQ, and QL conceived and designed the experiments. QL, JW, DH, and ML drafted and revised the manuscript. YY, YZ, and XL carried out the data collection. JW, DH, and YC carried out the data analysis and interpretation. DH, YQ, ML, and LH contributed reagents, materials, and analysis tools. All authors contributed to the article and approved the submitted version.

## Conflict of Interest

The authors declare that the research was conducted in the absence of any commercial or financial relationships that could be construed as a potential conflict of interest.
