# Supplementary material for: Excessive Neutrophils and Neutrophil Extracellular Traps in COVID-19
Source: Front Immunol. 2020 Aug 18;11:2063. doi: 10.3389/fimmu.2020.02063 (PMC7461898; doi:10.3389/fimmu.2020.02063)
Supplement: Supplementary file 1 [file Data_Sheet_1.doc]

**Supplemental files**

**Supplemental materials**

To make CT values better comparable between patients, we standardized the values to z-scores (“CTz”). Specifically, the z-score of CT value j of patient i is given by , where is the corresponding original CT value, is the mean of the CT values for patient i and is the standard deviation of the CT values for patient i. After the transformation, CTz values are approximately normally distributed and on the same scale (Supplement Figure 1A).

The neutrophil count distribution was skewed with a heavy tail towards high cell counts. After a log-transformation, neutrophil counts were approximately normally distributed (Supplement Figure 1B, left). Lymphocyte counts covered a much smaller range and were approximately normally distributed without transformation (Supplement Figure 1B, right).

To quantify the dependency of CTz on neutrophil and lymphocyte counts, we implemented linear regression in Stan via R-package rstanarm.^1-2^ In the notation of the linear models for CTz values as functions of neutrophils and lymphocytes were, respectively, CTz ~ (log(neutrophils)|severity) and CTz ~ (lymphocytes|severity).^3^ Models were treated formally as generalized linear models with the identity link function and Gaussian noise.
 The following priors were used: for the intercepts Gaussian priors with location 0 and scale 10, for the standard deviation an exponential with rate 1, and for the covariance a decov prior with regularization, concentration, shape, and scale all set to 1.
 Convergence of Markov Chain Monte Carlo (4 chains, each with 1000 steps warm-up and 1000 steps sampling) was checked by inspection of Gelman-Rubin parameter R, which was close to one for all considered models, typically < 1.01. Agreement of models with measurements was checked by visual posterior predictive checks (Supplement Figure 1C). Model ability to generalize was tested by approximate leave-one-out cross-validation, where all Pareto k estimates were good (k < 0.5).^4^

**Supplement Figure 1**


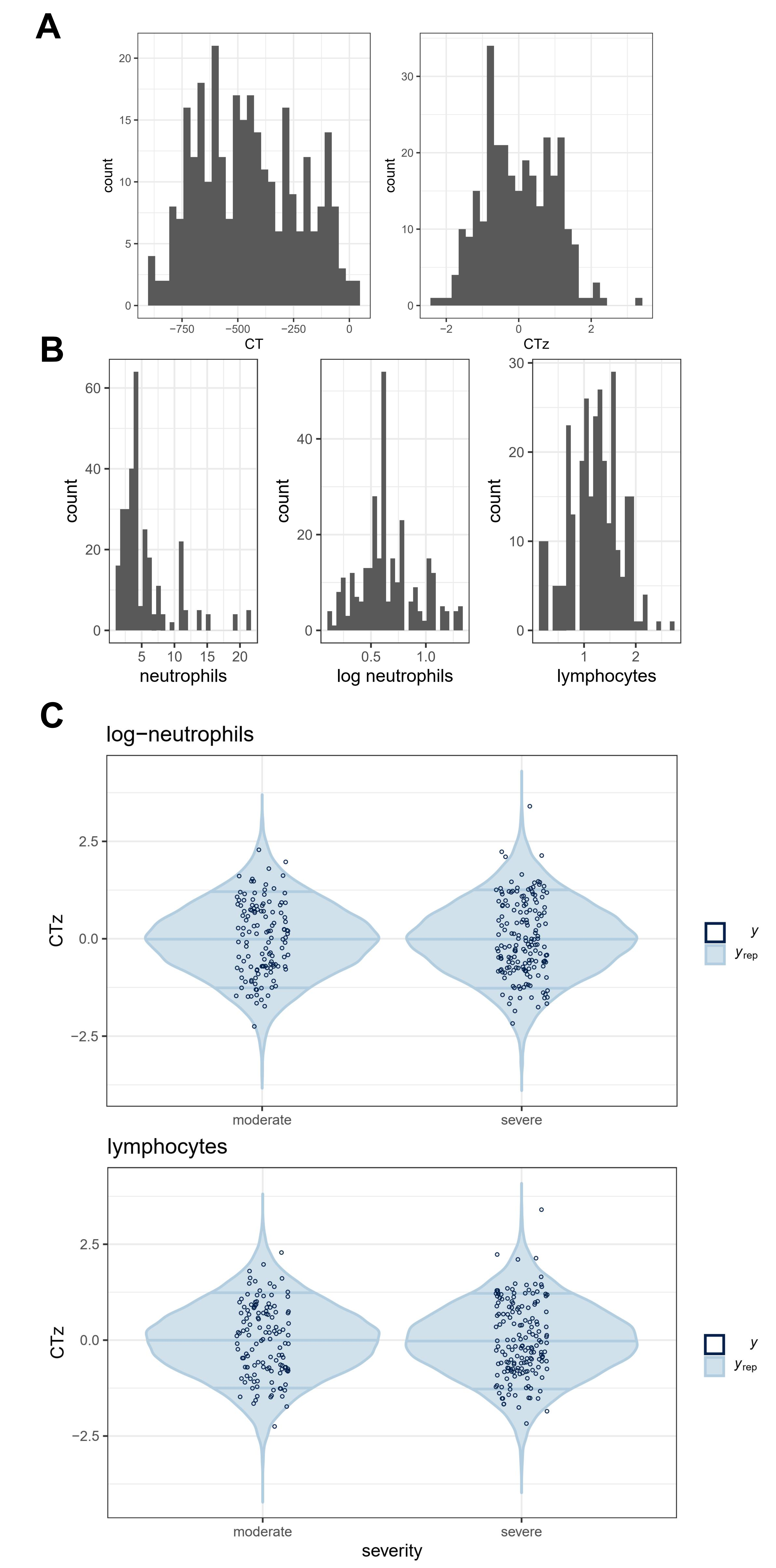


**Supplement Figure 1. Standardizing the values to z-scores (“CTz”) to make CT values better comparable between patients**

1. Histograms of original CT values of 23 patients (left), and after standardization to z-scores CTz (right).
2. Histograms of cell counts for 23 patients. Left: neutrophil counts; center: log-transformed neutrophil counts; right: lymphocyte counts.
3. Comparison of observed CTz values (points) and predictions by model (violins) for moderate and severe cases in both neutrophils (top) and lymphocytes (bottom).

**Reference**

1. Carpenter B, Gelman A, Hoffman MD, et al. Stan: A probabilistic programming language. Journal of Statistical Software 2017; 76(1).
2. Goodrich B, Gabry J, Ali I, et al. rstanarm: Bayesian applied regression modeling via Stan. R package version 2.17.4. URL: http://mc-stan.org/
3. Bates D, Mächler M, Bolker B, et al. Fitting linear mixed effects models using lme4. Journal of Statistical Software 2015; 67(1):1–48.
4. Vehtari A, Gelman A and Gabry J, et al. Practical bayesian model evaluation using leave-one-out cross-validation and waic. Statistics and Computing 2017; 27 (5): 1413–1432. URL: http://dx.doi.org/10.1007/s11222-016-9696-4

**Supplement Figure 2**


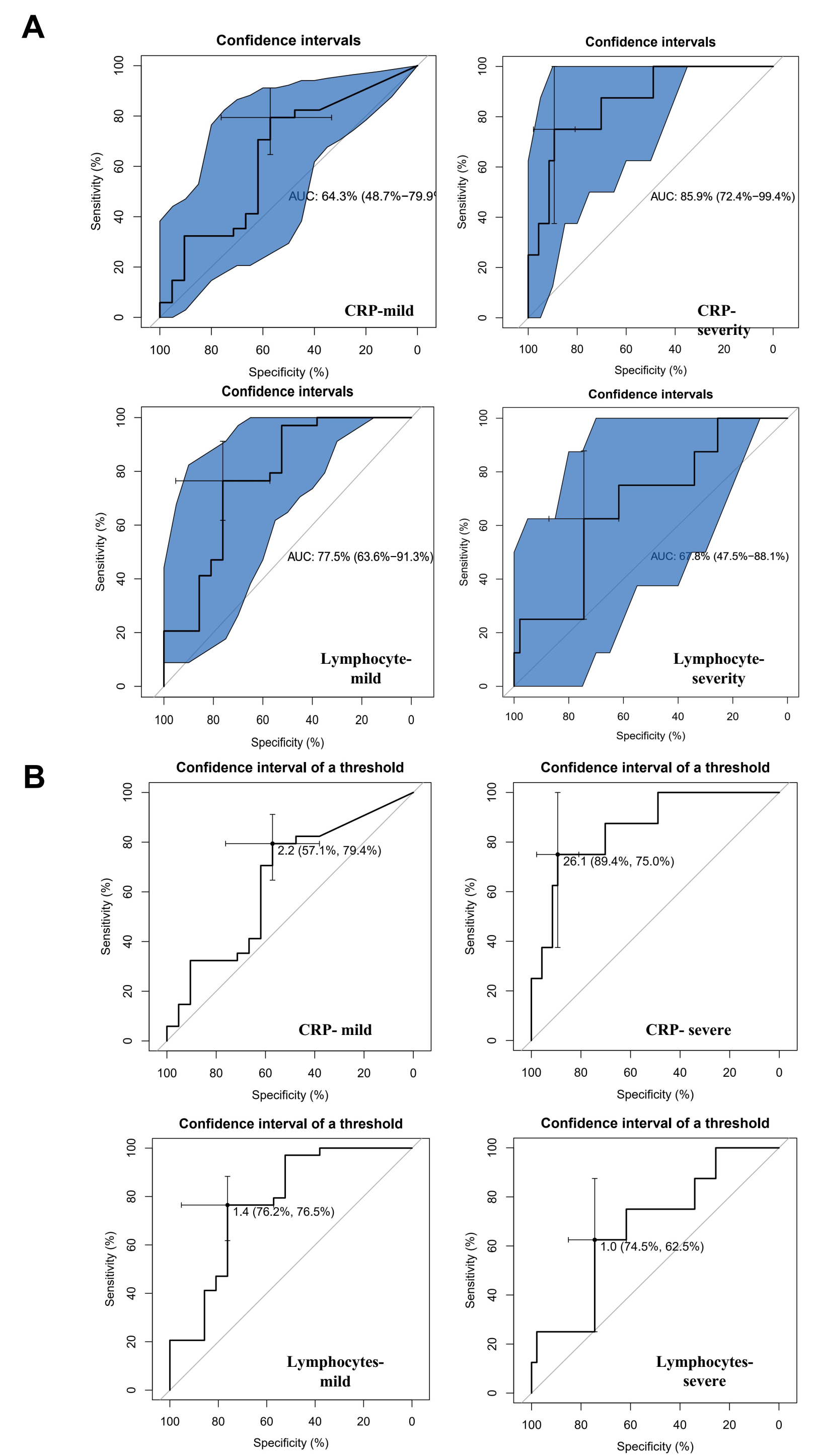


**Supplement Figure 2. ROC Curve and Cutoff Analysis**

(A) ROC curves and AUC were calculated for two selected parameters, CRP levels and lymphocyte counts, by using R package “pROC”. (B) The cut-off values were calculated from the corresponding ROC curves.

**Table S1 NETs associated proteins**

| **Metabolic enzymes** | **Structural Proteins** | **Nucleosome-Associated Proteins** | **An-microbial Related Proteins** | **Chaperone/**  **Support Proteins** | **Peroxisomal enzymes** | **Other enzymes / Not Classified** |  |
| --- | --- | --- | --- | --- | --- | --- | --- |
|  |
|  |
| TALDO1 | CORO1A | LYZ | LTF | ANXA1 | PRDX2 | [PRTN3](https://www.genecards.org/cgi-bin/carddisp.pl?gene=PRTN3&keywords=Proteinase,3) |  |
| TKT | ACTA2 | ANXA5 | SERPINB | ANXA3 | RETN | PLBD1 |  |
| GPI | ARPC1B | HIST1H4A | LCN2 | HSPA8 | CAT | [CHI3L1](https://www.genecards.org/cgi-bin/carddisp.pl?gene=CHI3L1&keywords=Chitinase-3-like,protein,1) |  |
| ALDOA | PFN1 | HIST3H3 | CAMP | HSPA1A | PADI3 | [MMP8](https://www.genecards.org/cgi-bin/carddisp.pl?gene=MMP9&keywords=Matrix,metalloproteinase,8) |  |
| TPI1 | MYL6B | ANP32A | ELANE | HSPA1L | FTH1 | PPIB |  |
| ENO1 | GSN | NAA38 | PSMA1 | MMP9 | SOD1 | SERPINA3 |  |
| PGK1 | ACTN4 | S100A4 | MPO | HSPE1 | SH3BGRL3 | [QSOX1](https://www.genecards.org/cgi-bin/carddisp.pl?gene=QSOX1&keywords=Quiescin,Q6,sulfhydryl,oxidase,1) |  |
| GAPDH | MSN | ARHGDIB | **HNE** | PPIA | GSTP1 | [SERPINA1](https://www.genecards.org/cgi-bin/carddisp.pl?gene=SERPINA1&keywords=Serpin,A1) |  |
| LDHB | ACTR3 | H2A | PADI3 | PRDX1 | **PADI4** | CTSC |  |
| LDHA | CAPZA1 | H2B | UBA52 | SET |  | HCK |  |
| PGAM1 | ACTB | H3 | CTSG | ANXA4 |  | CLEC4E |  |
| MDH1 | ACTN1 | H4 | PGLYRP4 | ANXA6 |  | FTL |  |
| MDH2 | FLNA | MNDA | EPX | HSPA2 |  | YWHAG |  |
| TKTL | MYH | CLC | AZU | HSPA5 |  | YWHAE |  |
| PPIase | LCP1 | ECP | LYZ |  |  | YWHAB |  |
| DKFZp686B04128 | KRT-10 | HMGB2 | S100A4 |  |  | AZGP1 |  |
| RAC2 | VIM | HMGN2 | S100A12 |  |  | C3 |  |
| NCF2 | CFL-1 | HP1BP3 | BPIB2 |  |  | CGA |  |
|  | TMSB4X |  | ELA2 |  |  | [CRISP](https://www.genecards.org/cgi-bin/carddisp.pl?gene=CRISP3&keywords=Cysteine-rich,secretory,protein) |  |
|  | LSP1 |  | PR3 |  |  | HPX |  |
|  | TPM2 |  | DEFA-1/3 |  |  | IQGAP1 |  |
|  | VCL |  | S100A8 |  |  | [MUC5B](https://www.genecards.org/cgi-bin/carddisp.pl?gene=MUC5B&keywords=Mucin,5B) |  |
|  |  |  | S100A9 |  |  | A1BG |  |
|  |  |  | GRN |  |  | CAP1 |  |
|  |  |  | LL-37 |  |  | GMFG |  |
|  |  |  | TREM1 |  |  | BASP1 |  |
|  |  |  | C1QB |  |  | ITGAM |  |
|  |  |  | C1QC |  |  | LGALS9 |  |
|  |  |  |  |  |  | CCL7 |  |
|  |  |  |  |  |  | CCL8 |  |
|  |  |  |  |  |  | CEACAM1 |  |
|  |  |  |  |  |  | SIGLEC14 |  |
|  |  |  |  |  |  | **CXCL16** |  |
